# Supplementary material for: Effect of Drought and Methyl Jasmonate Treatment on Primary and Secondary Isoprenoid Metabolites Derived from the MEP Pathway in the White Spruce Picea glauca
Source: Int J Mol Sci. 2022 Mar 30;23(7):3838. doi: 10.3390/ijms23073838 (PMC8998179; doi:10.3390/ijms23073838)
Supplement: Supplementary file 1 [file ijms-23-03838-s001.zip › ijms-1629249-supplementary.pdf]

Supplementary Material

Treatment															
**Drought time course (day)**	0	1	2	3	4	5	6	7	8	9	10	11	12	13	14
**MeJA time course (day)**					0	1	2	3	4	5	6	7	8	9	10
						**First Sampling**									**Second Sampling**

Figure S1: Time course of the experiment. Overall, the experiment took 14 days. Drought was begun at day 0, with MeJA application occurring on day 4. First sampling occurred on day 5 of the drought treatment, one day after MeJA application. The second sampling occurred on the 14th day of the drought treatment, 10 days after MeJA application.

Table S1: Effect of application of drought (D) and MeJA (M), separately and combined (D+M), on amounts of monoterpenes emitted (ng g^{-1}DW h^{-1}) of each compositional group at first and second sampling points. Results are given as mean ± SEM. C, control

First sampling	Non-oxygenated	Oxygenated	Induced
C	1052 ± 516	180 ± 94	6 ± 3
D	763 ± 167	586 ± 231	9 ± 3
M	6051 ± 2389	2571 ± 609	5787 ± 1077
DM	1532 ± 372	2249 ± 1043	875 ± 284
**Second sampling**	**Non-oxygenated**	**Oxygenated**	**Induced**
C	195 ± 46	25 ± 13	19 ± 2
D	559 ± 241	112 ± 58	24 ± 7
M	4774 ± 3410	1979 ± 1523	160 ± 68
DM	1281 ± 326	278 ± 70	40 ± 10

Table S2: List of qRT-PCR primers used in this study. Primer sequences are listed from 5′->3′ with F for forward and R for the reverse primer.

PRIMER	SEQUENCES
DXS 1F	AAGGGAGCACGAGGTAATAATAAC
DXS 1R	CGATGTAGTGGTCAGGAAGAAC
DXS 2A-1F	GTTGACAGTGGAGGAAGG
DXS 2A-1R	ATTATGGTAGCCGCAATATG
DXS 2B-1F	TGGTTGTAATGGCTCCTTC
DXS 2B-1R	GGGTTCCTTTGTTGTTTGG
DXR-F	ATCTTGCTTATTCTGCTGGTAG
DXR-R	TGGTCATAGTGTATTATCTCTTCC
UBI-F	GTTGATTTTTGCTGGCAAGC
UBI-R	CACCTCTCAGACGAAGTAC
